# Antiphospholipid Syndrome and the Neurologist: From Pathogenesis to Therapy

**DOI:** 10.3389/fneur.2018.01001

**Published:** 2018-11-26

**Authors:** Thomas Fleetwood, Roberto Cantello, Cristoforo Comi

**Affiliations:** ^1^Section of Neurology, Department of Translational Medicine University of Eastern Piedmont, Novara, Italy; ^2^Interdisciplinary Research Centre of Autoimmune Diseases University of Eastern Piedmont, Novara, Italy

**Keywords:** APS, antiphospholipid syndrome, aPL, antiphospholipid antibodies, neurological manifestations, pathogenic mechanisms, therapy

## Abstract

Antiphospholipid syndrome (APS) is an autoimmune antibody-mediated condition characterized by thrombotic events and/or pregnancy morbidity in association with persistent positivity to antiphospholipid antibodies (aPL). The nervous system is frequently affected, as intracranial vessels are the most frequent site of arterial pathology. Over the course of years, many other neurological conditions not included in the diagnostic criteria, have been associated with APS. The pathogenic mechanisms behind the syndrome are complex and not fully elucidated. aPL enhance thrombosis, interfering with different pathways. Nevertheless, ischemic injury is not always sufficient to explain clinical features of the syndrome and immune-mediated damage has been advocated. This may be particularly relevant in the context of neurological complications. The reason why only a subgroup of patients develop non-criteria nervous system disorders and what determines the clinical phenotype are questions that remain open. The double nature, thrombotic and immunologic, of APS is also reflected by therapeutic strategies. In this review we summarize known neurological manifestations of APS, revisiting pathogenesis and current treatment options.

## Introduction

Antiphospholipid syndrome (APS) is an autoimmune antibody-mediated disorder defined by the occurrence of thrombosis and/or pregnancy morbidity in presence of persistent antiphospholipid antibodies (aPL) (1). The estimated incidence of APS is approximately 5 cases every 100,000 subjects/year with a prevalence of 40–50 every 100,000 subjects (2). The diseases can occur alone (primary APS) or in the context of other autoimmune conditions, in particular systemic lupus erythematosus (SLE), Sjögren's syndrome and rheumatoid arthritis (secondary APS). Veins, arteries and small vessels can all be affected by thrombosis, with deep veins of the legs and intracranial arteries being the most common sites of venous and arterial thrombosis, respectively. Pregnancy morbidity includes embryonic losses, fetal death, and premature birth. The diagnosis is made according to the updated international Sydney consensus criteria (Table 1) (1). However, patients with persistent aPL may present with clinical manifestations not included in the criteria (the so called “non-criteria” symptoms), among which thrombocytopenia, hemolytic anemia, cardiac valve disease, renal microangiopathy, livedo reticularis, and neurologic disturbances other than ischemic cerebrovascular accidents (CVA) (3).

**Table 1:** Revised classification criteria for APS [adapted from Miyakis et al. (1)].

**Clinical criteria (one or more of the following):**
**VASCULAR THROMBOSIS**
•One or more clinical episodes of arterial, venous, or small vessel thrombosis, in any tissue or organ.
**PREGNANCY MORBIDITY**
•One or more unexplained deaths of a morphologically normal fetus at or beyond the 10th week of gestation •One or more premature births of a morphologically normal neonate before the 34th week of gestation because of eclampsia or severe preeclampsia (placental insufficiency). •Three or more unexplained consecutive spontaneous abortions before the 10th week of gestation.
**LABORATORY CRITERIA (ONE OR MORE OF THE FOLLOWING):**
1. Lupus anticoagulant (LA) in plasma, on two or more occasions at least 12 weeks apart, detected according to the guidelines of the International Society on Thrombosis and Haemostasis. 2. Anticardiolipin antibody (aCL) of IgG and/or IgM isotype in serum or plasma, present in medium or high titer (i.e., >40 GPL or MPL, or >the 99th percentile), on two or more occasions, at least 12 weeks apart, measured by a standardized ELISA. 3. Anti-beta-2-glycoprotein-I antibody (anti-β2-GPI) of IgG and/or IgM isotype in serum or plasma (in titer >the 99th percentile), present on two or more occasions, at least 12 weeks apart, measured by a standardized ELISA.

In this paper we summarize current concepts regarding the pathogenesis of APS, reviewing neurological clinical features and related therapeutic implications.

## Pathogenesis of APS: thrombosis and other mechanisms

**Figure 1:**
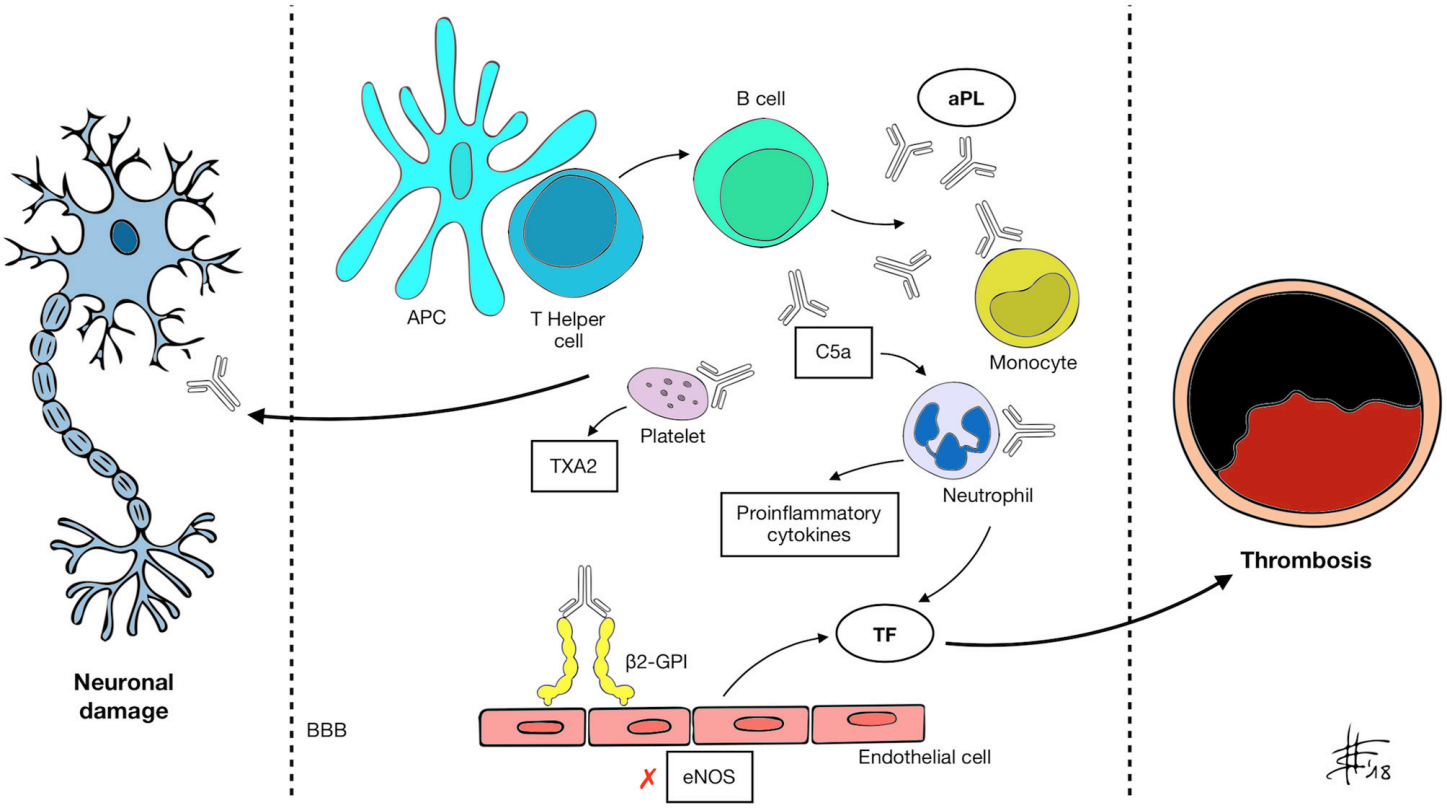
APC, antigen presenting cell; aPL, antiphospholipid antibody; β2-GPI, β2 glycoprotein-I; BBB, blood-brain barrier; C5a, activated complement 5 fraction; eNOS, endothelial nitric oxide synthase; TXA2, thromboxane A2; TF, tissue factor. A mechanism of molecular mimicry may underlie B cell activation by the APC-T helper system and subsequent aPL production in susceptible individuals. Through recognized antigens (mainly β2-GPI) aPL interact with different cell types and activate the classical complement pathway, inducing expression of pro-inflammatory cytokines, enhancing platelet adhesiveness and aggregation, impairing vascular tone by inhibiting eNOS and increasing the expression of TF. These changes determine a prothrombotic state and increase the risk of vascular events. aPL also appear to be able to recognize and target nervous system antigens, possibly through the disruption of the BBB. Neurological manifestations of APS may be determined by both mechanisms.

Clinical manifestations in APS are associated with aPL presence. aPL, namely lupus anticoagulant (LA), anti-cardiolipin (aCL), anti-β2-glycoprotein-I (anti-β2-GPI), are an heterogeneous group of auto-antibodies directed against phospholipid binding proteins (4). The current hypothesis is that susceptible individuals develop aPL upon exposure to an external trigger, such as an infective agent, through a mechanism of molecular mimicry (2). β2-GPI, one of the main antigens recognized by aPL, is a plasma protein composed of five domains (I–V) (5). Circulating β2-GPI presents a circular form, in which epitopes present in domain I and recognized by B-cells are hidden. Upon binding to an anionic phospholipid surface, β2-GPI undergoes a conformational change exposing the cryptic epitopes (6). Oxidative stress seems to enhance the immune reaction, leading to the formation of disulfide bonds in domain I that further increase its immunogenic potential (7). Prevalence of aPL in the general population is around 1–5% (8) but only a minority of subjects develops APS, suggesting that the presence of autoantibodies alone is not sufficient to cause pathology. Following aPL formation, a “second hit” thus is necessary. It has been demonstrated that β2-GPI only binds its ApoE receptor 2 (ApoER2) when vessel endothelial cells become activated (9), inducing dimerization of the receptor and activation of intracellular signaling. A number of conditions that increase oxidative stress, including infection, malignancy, pregnancy and smoking, may act as triggers for endothelium priming (10, 11). The mechanisms through which aPL enhance thrombosis is complex and in part still to be understood. Different mediators are likely to be involved, among which endothelium, platelets, complement, and innate immune systems (9) (Figure 1). First, aPL stimulate the expression of proadhesive, procoagulant, and proinflammatory molecules. Tissue factor (TF) is a key component of the extrinsic coagulation pathway, implicated in the activation of thrombin, that becomes exposed upon vessel injury. aPL enhance surface expression of TF on endothelial cells by binding to annexin A2 and toll-like receptor 4 (TLR4) and activating the nuclear factor κB (NF-κB) signaling pathway (12–14). aPL also seem to interact directly with monocytes and neutrophils, inducing mitochondrial dysfunction and subsequent expression of TF and proinflammatory tumor necrosis factor α (TNF-α) (15). Annexin A5 is a protein involved in many biological processes. On endothelial cells it binds to phosphatidylserine molecules, forming a shield that inhibits the activation of procoagulant complexes. *In vitro* studies have shown that aPL binding to annexin A5 disrupts the shield leading to thrombosis (16). Endothelial cells also contribute to modulate the activity of vessel wall muscular cells through the production of nitric oxide by endothelial nitric oxide synthase (eNOS). In murine models, aPL inhibit the activity of eNOS (17). This can lead to impaired regulation of vascular tone, increase in superoxide and peroxynitrite, and cell adhesion (18, 19). Confirming this hypothesis, APS patients have reduced plasma levels of nitric oxide compared to controls (20). Platelets play a pivotal role in thrombus formation. β2-GPI binds to the von Willebrand factor (vWF) receptor glycoprotein Ibα and to the ApoER2 on the platelet surface inducing the release of thromboxane A2 and enhancing aggregation and adhesiveness (21). Interestingly, binding of aPL to platelet membrane phospholipids leads to activation and possible dysregulation of serotonin metabolism, which could be involved in the pathogenesis of aPL-mediated migraine (22). Some authors have also identified anti-platelet antibodies in the setting of APS (23). aPL can activate the classical complement pathway, inducing production of C5a, which in turn can bind to neutrophils and stimulate the expression of TF (24). Previous studies have also suggested that aPL impair thrombolysis by interfering with tissue plasminogen activator (tPA) and plasmin (25). Despite the amount of evidence gathered over the course of years, thrombosis alone is perhaps not sufficient to explain all of the clinical effects of aPL. Direct binding of aPL to nervous system antigens has been proposed to explain some neurologic manifestations of APS, which may be mediated by inflammation and neurodegeneration (26, 27). An experimental model of APS (eAPS) can be obtained by immunizing normal mice with β2GPI, therefore inducing aPL and typical clinical features of APS (28). Interestingly, in eAPS mice, long-term exposition to aPL also leads to behavioral hyperactivity and decline in cognitive performances (29, 30), which resolve after elimination of aPL through ultraviolet irradiation (26). A proposed mechanism of neuronal injury focuses on the disruption of the blood-brain barrier (BBB), secondary to diffuse endothelial dysfunction caused by aPL binding (31). Katzav et al. have demonstrated impaired integrity of the BBB and accumulation of aPL in cortical and inhibitory hippocampal neurons of immunized mice (32), which may be linked to behavioral changes and cognitive impairment. Furthermore, in a previous study eAPS mice displayed higher brain levels of proinflammatory TNF-α and prostaglandin E (PGE) and lower levels of thrombin inhibitors compared to controls (33). Treatment with aspirin and enoxaparin ameliorated concentrations of TNF-α, PGE and thrombin inhibitors, as well as behavioral patterns. An interesting correlation between coagulation and autoimmunity has been investigated in eAPS mice carrying the factor V Leiden mutation (FVL), either in homozygosis (FVL^Q/Q^) or in heterozygosis (FVL^Q/−^). Induced aPL levels were higher in FVL^Q/Q^ mutated mice compared to FVL^Q/−^ and controls, as well as the burden of behavioral and cognitive impairment and of neurodegenerative changes on histological examination (34). It is known from previous studies that aPL have the potential to bind to myelin, brain ependyma and choroid epithelium epitopes in the animal model (35). A direct interaction between aPL and specific, yet still unidentified, basal ganglia epitopes, may lead to the development of movement disorders in APS patients. Supporting this hypothesis Dale et al. in 2011 demonstrated binding of IgG from the serum of pediatric APS patients with chorea to neuronal cell-surface antigens of cultured neuronal cells with dopaminergic characteristics (36). Furthermore it has been shown that aPL can impair GABA receptor activity and induce depolarization of synaptoneurosomes, disrupting neuronal function by acting on nerve terminals (37, 38), with possible implications in APS-associated epileptogenesis. Energetic dysfunction in neuronal cells and altered neurotransmission may also play a role in APS pathology, since it has been demonstrated that aPL from patients with neurological involvement also bind to adenosine triphosphate (ATP) (39).

## Neurological manifestations of APS

The nervous system is a major target of APS. This appeared clear since the first description of the syndrome by Hughes in 1983 in which he described CVA and transverse myelitis (40). Although thrombotic damage has been advocated to explain many neurologic manifestations, direct immune-mediated processes may also be involved (41). Neurologic symptoms have been therefore classified in thrombotic and non-thrombotic according to the supposed primary pathogenic mechanism (Table 2) (42). The reason why some patients develop neurological symptoms is unknown as well as what determines the site of lesion (e.g., central vs. peripheral nervous system) and the clinical phenotype. Possible explanations may be related to antibody subtypes, individual susceptibility or even genetic predisposition.

**Table 2:** Neurological manifestations of APS.

**Thrombotic**	**Non-thrombotic**
•Acute ischemic stroke •Transient ischemic attack (TIA) •Cerebral venous thrombosis (CVT) •Sneddon's syndrome •Reversible cerebral vasoconstriction syndrome (RCVS)	•Headache •Seizures and epilepsy •Movement disorders •Multiple sclerosis-like disease •Transverse myelitis •Cognitive impairment and dementia •Neuropsychiatric symptoms •Peripheral neuropathy •Autonomic dysfunction

### Cerebrovascular disease

Acute ischemic stroke and transient ischemic attack (TIA) are the most common manifestations of arterial pathology in APS (2). According to the Euro-Phospholipid Project Study Group the cumulative prevalence of stroke and TIA in APS patients are 19.8 and 11.1%, respectively, (43). On the other hand, aPL may be detected in up to 13.5% of stroke patients (44), with higher rates in young subjects. Moreover, age at onset in aPL-positive patients is anticipated. A previous study on 128 patients with CVA and aPL showed a mean age was 46 years (45). It has been suggested that APS accounts for over 20% of cases in the young population (46). A 2015 systematic review of 43 studies showed that the presence of aPL in subjects under the age of 50 increased the risk for thrombotic cerebrovascular events by 5.48-fold (47). The association between aPL and stroke incidence in older patients is less clear, due to the higher prevalence of other vascular risk factors (48). Clinical manifestations depend on the site and entity of the lesion. In a sequence of 110 SLE and non-SLE aPL-positive patients undergoing neuroradiological evaluation, the most common finding was large infarcts (22%), followed by white matter changes (17%), small cortical infarcts (10%), and lacunar infarcts (9%) (49). In a cohort of 55 APS patients, 25 of which suffered an ischemic stroke, the most common site of occlusion was the middle cerebral artery (31%) (50). *In situ* thrombosis is thought to be the most frequent pathogenic mechanism, whereas in other cases, cardiac embolisms can arise from involvement of the cardiac valvular apparatus, with thickening of valve leaflets by deposition of immune complexes (Liebman-Sacks endocarditis) (51). Besides stem or branch occlusion of intracranial arteries, a vasculitis-like pattern, with multiple sites of narrowing and dilation has also been described through arteriography (52). Alterations of extracranial arteries appear to be less frequent (52), though early atherosclerosis seems to affect APS patients (53), and some authors have proposed a direct role of the autoantibodies through oxidative damage (54). Although rare, cerebral venous thrombosis (CVT) can complicate APS or, in some cases, be the presenting symptom (55). Presence of aPL in non-SLE patients with CVT has been reported in previous studies (56). aCL positivity may be found in 7–22% of patients (51). APS accounts for ~6–17% of all CVT cases, being one of the most frequent prothrombotic conditions associated (57). Other vascular conditions have also been reported in association with APS, including Sneddon's syndrome and reversible cerebral vasoconstriction syndrome (RCVS). Sneddon's syndrome is a rare non-inflammatory thrombotic vasculopathy, characterized by livedo reticularis and recurrent cerebral infarctions (53). Other clinical manifestations include headache, seizures and cognitive decline (58). Details of the pathogenetic mechanisms are still to be clarified but non-inflammatory thrombotic vasculopathy is seen in medium- and small-sized arteries in the brain and skin (59). Indeed, neuroradiological findings show that leukoaraiosis and small lacunar infarcts are more common than infarcts in the territory of large cerebral arteries (60). Reversible cerebral vasoconstriction syndrome (RCVS) is a neurological disorder marked by severe headaches variably associated with seizures, ischemic stroke, and subarachnoid hemorrhage (61). The cause has been related to a possible disturbance in the regulation of cerebrovascular tone (62).

### Headache

Headache, specifically in the form of migraine, is the most common neurologic symptom of APS patients, with an estimated prevalence of 20.2% (43). Indeed, aPL antibodies are more frequently found in migraineurs than in age-matched controls (63). However, given its high prevalence in the general population, it is difficult to establish whether APS is a risk factor for developing migraine or if this represents a comorbid condition. Previous CVA represent a strong risk factor for developing migraine. However, not all patients have a positive history for stroke or TIA, which suggests non-ischemic mechanisms (64). On the contrary some authors have found long-standing migraine to be a risk factor for stroke in APS patients (65). Further studies are necessary to elucidate such interplay. Notably, APS-associated migraine may be difficult to control with classic analgesic regimens (66).

### Seizures and epilepsy

APS seems to confer a higher risk of developing seizures, with an estimated prevalence of ~8% (67), which may further increase in APS secondary to SLE (68). All forms of epilepsy may be seen, including subclinical forms, determined by the presence of abnormal electroencephalography findings alone (46). It has been suggested that 20% of idiopathic juvenile epilepsy cases may be associated with aPL (69). According to existing studies, previous CVA have been identified has the most solid risk factor for developing seizures (68). It is therefore easy to speculate that the most plausible pathogenic mechanism is ischemic damage to brain tissue, leading to the formation of cortical epileptogenic foci (64). However, seizures may also develop in structurally normal brains, suggesting an antibody-mediated mechanism (70).

### Movement disorders

Movement disorders are a possible, though rare, neurological manifestations in the setting of APS. Chorea in particular occurs in 1.3–4.5% of patients (71), and may be the first symptom of the syndrome (72, 73). Other less frequently reported conditions include parkinsonism (corticobasal-like syndrome and progressive supranuclear palsy phenotype), dystonia, ballismus, paroxysmal dyskinesias, tremor, tic, myoclonus, cerebellar ataxia (41). Mixed clinical presentations have also been described (74). The pathogenesis of movement disorders in the setting of APS is matter of debate. On the one hand, cerebral infarctions and white matter changes on MRI suggest a thrombo-occlusive mechanism, which could account for the majority of cases (51, 75). On the other, immune-mediated attack against basal ganglia epitopes has been suggested and reported in some cases (41). No specific correlations between the type of antibody and clinical features have been reported (41). Nevertheless, it is likely that genetic predisposition might partially explain phenotypic variability, in analogy to what was reported in Parkinson's disease (76).

### Multiple sclerosis-like disease

Overlap of clinical and laboratory findings of multiple sclerosis (MS) and SLE have been described long ago, leading to the proposal of a hybrid condition named “lupoid sclerosis” (77, 78). Diagnostic criteria, as well as the role of anti-nuclear antibodies (ANA) and aPL are still matter of debate (79). Several studies have provided different estimates of aPL prevalence in definite MS patients, ranging from 2 to 88% (80), with higher titers observed during exacerbations of the disease (81). aCL and anti-β2GPI appear to be more prevalent compared to LA, although the latter have been studied to a lesser extent and the real prevalence remains to be clarified (80). Some authors have suggested that aPL may alter the integrity of the BBB and facilitate the access of immune cells to the central nervous system (CNS) compartment (81, 82). APS can also present with symptoms resembling MS, including visual, sensitive or motor deficits with a relapsing remitting course, with similar MRI T2 lesions (83–87). This condition is referred to as MS-like disease (64). Indeed, the differential diagnosis between these two conditions may be challenging. Acute onset of atypical MS symptoms, coexistence of other typically APS-related neurological manifestations (for example headache or epilepsy), connective tissue-like features or a history of thrombosis, pregnancy morbidity should orientate toward APS (88, 89). Neuroimaging studies may also help in the differential diagnosis. APS lesions on MRI are smaller, frequently localized in the subcortical area, are stable over time and may also improve with anticoagulation therapy (80, 90). Normal cell count and absence of oligoclonal bands on CSF analysis also suggests APS (91). Some authors have suggested that aPL may alter the integrity of the BBB and facilitate the access of immune cells to the central nervous system (CNS) compartment (81, 82).

### Transverse myelitis

Transverse myelitis (TM) is an inflammatory condition affecting the gray and white matter of the spinal cord (92). Symptoms include motor and sensory level deficits and sphincter abnormalities. Estimated prevalence in APS is around 0.4–4% (71, 93). Although the exact pathogenesis is unsure, vasculitis and arterial thrombosis resulting in ischemic cord necrosis have been suggested (89). Furthermore, APS has been described in overlap with neuromyelitis optica spectrum disorder (NMOSD) (94, 95), another autoimmune condition characterized by recurrent episodes of optic neuritis and longitudinally extensive transverse myelitis (that is, extended over 3 or more spinal cord segments) and positivity for anti-aquaporin-4 (anti-AQP4) or anti-myelin oligodendrocyte glycoprotein (anti-MOG) (96). Therefore, some authors suggest screening APS patients presenting with optic neuritis or myelitis for NMO-associated autoantibodies (95).

### Cognitive impairment and dementia

A high percentage of patients with primary APS (which may reach 42–80%) develop some degree of cognitive impairment, usually with a subcortical pattern (64, 97). In some cases, deficits may even precede the diagnosis of APS, as demonstrated in a study by Jacobson et al. on aPL-positive non-elderly subjects who displayed differences in executive functioning, verbal learning and memory, and visuospatial ability compared to age- and education-matched controls (98). Dementia frequency has been estimated around 2.5% in APS patients (71). MRI studies have shown a high burden of white matter lesions in APS patients with cognitive impairment (99) resembling multi-infarct dementia. However, vascular damage may not be the only mechanism. Findings of degenerative rather than multi-infarct dementia have also been described in aPL-positive elderly subjects (100). Other studies and a meta-analysis have underlined a strong association with aCL antibodies (101). Furthermore, some animal models showed that cognitive dysfunction can be induced by intraventricular injection of neuronal-binding antibodies from APS patients (26), while other failed to demonstrate an association with ischemic lesions (102). Such findings support the idea of a direct effect of aPL on congnition. aPL-mediated dysregulation of the dopaminergic system has also been proposed (103). Given the clinical overlap, MS-like disease should also be considered in the differential diagnosis.

### Neuropsychiatric symptoms

Psychiatric symptoms, including psychosis, mania, depression, bipolar disorders, obsessive–compulsive disorders, and schizophrenia have been described in APS patients (64). Older age, cerebral lesions, and triple aPL positivity (i.e., anti-β2-GPI, aCL, and LA) are considered risk factors (104). A high prevalence of aPL has been described in patients with psychosis, though such finding must be interpreted carefully as antipsychotic medications are though to induce aPL and aCL in particular (66).

### Peripheral neuropathy

A small study by Santos et al. in 2010 has investigated the involvement of the peripheral nervous system in APS (105). The most frequent findings were sensori-motor neuropathy and isolated carpal tunnel syndrome. Most patients were asymptomatic and showed no sign of pathology upon physical examination. The underlying pathogenic mechanism is not clear, and may be linked to thrombosis of the vasa nervorum, vasculitis or even targeting of lipidic components of myelin by aPL. Reports of Guillain-Barré syndrome in APS patients support this hypothesis (106).

### Autonomic dysfunction

Several reports exist of autonomic dysfunction in the context of APS. A 2017 work by Schofield describes the clinical findings in 22 patients with autonomic dysfunction as the initial symptom of APS (107). Manifestations included postural tachycardia syndrome, neurocardiogenic syncope, inappropriate sinus tachycardia, labile hypertension, complex regional pain syndrome, severe gastrointestinal dysmotility, and neurogenic bladder, with 45% of subjects presenting more than one disorder. Reduction in autonomic and sensory small fibers, assessed by skin punch biopsy was widespread among subjects. Ten patients (45%) subsequently developed arterial thrombosis, of which 8 (36%) presented with stroke, TIA, or amaurosis fugax. Pathogenesis of small fiber dysfunction may derive either from microthrombosis or from direct antibody binding to neuronal epitopes leading to nerve dysfunction. The latter hypothesis is supported by reported improvement of symptoms with immune modulatory therapy (107).

## Treatment: current concepts

To date, antithrombotic therapy represents the cornerstone of APS management. This is primarily based on vitamin K antagonists (VKA), generally warfarin (108). The high risk of thrombosis recurrence, which can be seen in 5–16% of subjects (109), warrants long-term anticoagulation. In the acute phase, ischemic cerebrovascular events are managed according to clinical standards as for patients without APS. Reports of intravenous thrombolysis in APS patients who develop acute ischemic stroke date back to 1997 (110). aPL-induced thrombocytopenia or prolongation of prothrombin time are issues to be considered before starting treatment (111). Primary endovascular thrombectomy may be considered as an alternative in these patients (112). Anticoagulation with warfarin and INR 2–3 is the most common strategy for secondary thromboprophylaxis (113). Risk stratification combining antibody profile and comorbidities could help to identify patients needing a more aggressive treatment but to date no model has been validated (3). Although no strong evidence exists in this sense, long-term antiplatelet therapy with low-doses aspirin may be beneficial in addition to warfarin (114). Recurrence of thrombosis frequently depends from inadequate anticoagulation (115), sometimes linked to aPL artifactual prolongation of prothrombin time. Nevertheless, in a series of 66 APS patients treated to a target INR of 3.5 a recurrence rate of 9.1/100 patient-years was detected, with recurrences often affecting the same vascular bed as the original thrombosis (116). Due to the lack of solid evidence, management of recurrences is not standardized and may include higher-intensity warfarin therapy (INR 3–4), the addition of low-dose aspirin or the use of low-molecular-weight heparin (3). Prophylactic treatment in asymptomatic aPL carriers is matter of debate. The risk of a first thrombotic event is likely liked to the presence of other concomitant factors (117–120). The use of low-dose antiplatelet therapy for primary prophylaxis is controversial, due to the lack of solid evidence of efficacy and some authors suggest management following guidelines for prevention of cardiovascular disease in the general population (3). Under specific conditions, immunosuppressive treatment may be an option in addition to antithrombotic therapy. The paradigm of this is represented by catastrophic APS (CAPS), a rare condition characterized by widespread small vessel thrombosis and subsequent multiorgan failure in the context of APS (2) with a mortality rate up to 37% (121). In the case of CAPS, aggressive treatment is recommended and consists of a “triple therapy” composed by anticoagulants plus glucocorticoids plus plasma exchange (PE) and/or intravenous immunoglobulins (IVIG) (122). Rituximab (RTX) is an anti-CD20 monoclonal antibody that depletes B-cells, currently used to treat several autoimmune diseases and hematologic malignancies. B-cells seem to play a pivotal role in the pathogenesis of APS (123). Several case reports describe the use of RTX in APS patients, suggesting a beneficial role in the management of hemolytic anemia (124), thrombocytopenia (125) (126), thrombotic microangiopathy (127). Despite no strong evidence, RTX may be used in patients with thrombotic relapses while on AVK and adequate INR, especially in the setting of APS secondary to SLE (128–130). No standard treatment exists for non-thrombotic neurological manifestations of APS and available evidence mostly derives from retrospective non-randomized trials or case reports (64). Besides cerebrovascular events, anticoagulation has proven effective in the treatment of conditions that are not primarily thrombotic, including migraine, transverse myelitis, and neuropsychiatric disturbances (131–133). Improvement of cognitive performance with anticoagulation therapy has also been described (46). Notably, response to anticoagulation may help distinguish APS from atypical presentations of CNS inflammatory disorders (80). The combined use of neuroleptics and antiplatelet or anticoagulant therapy, with or without steroids, can help control aPL-associated chorea (74, 134, 135). On the other hand the potential role of aPL-mediated damage provides the rational for the use of immunosuppressive therapy (136). Reports describe clinical remission of pediatric aPL-associated chorea achieved with mycophenolate mofetil, an immunosuppressant agent primarily used to prevent rejection in organ transplant induced (137), and IVIG (138). Steroids have been successfully used to treat psychotic symptoms in the context of APS (139). Furthermore, a 2013 non-randomized pilot study (RITAPS) demonstrated partial or complete remission of cognitive dysfunction in a small sample of patients treated with RTX (140).

### Future directions in the treatment of APS

#### Antithrombotic agents

Direct oral anticoagulants (DOACs) represent in interesting possible alternative to warfarin, especially considering the young age of many patients and the need to monitor INR with VKA. Furthermore, it has been suggested that the therapeutic effect of DOACs may go beyond anticoagulation and exert an anti-inflammatory and anti-angiogenic effect (141). Many case reports and case series have been published on the use of DOACs (mainly rivaroxaban) in APS, with heterogeneous results (142). However, only a few solid randomized controlled trials are available. In 2016 the RAPS trial by Cohen et al. failed to demonstrate the non-inferiority of rivaroxaban over warfarin in APS patients (143). Indeed, questions have been raised regarding thrombotic risk and safety issues related to DOACs, especially in triple-positive patients (144). Given the lack of solid evidence, the 15th International Congress on Antiphospholipid Antibodies Task Force on Antiphospholipid Syndrome Treatment Trends in 2017 did not support the use of DOACs instead of warfarin (142). Another trial by Pengo et al. on high-risk APS patients treated with rivaroxaban or warfarin was prematurely terminated because of the higher rate of thromboembolic events and major bleeding in the rivaroxaban group (145). Although other studies are currently ongoing investigating the effect of rivaroxaban and other DOACs, warfarin represents at the moment the only approved anticoagulant agent. Platelet activation and aggregation represents a further target for therapeutic agents. Glycoprotein IIb/IIIa (GPIIb/IIIa) receptor inhibitors are a relatively new class of antiplatelet drugs compared to aspirin, mainly used in the field of coronary disease. In murine models aPL-enhanced thrombosis can be inhibited by infusion of a GPIIb/IIIa antagonist monoclonal antibody and GPIIb/IIIa-deficient mice are resistant to aPL-mediated thrombosis (146, 147). Management of acute myocardial infarction in APS can represent a challenge due to the high risk of restenosis after angioplasty (148). Abciximab, a monoclonal antibody targeting GPIIb/IIIa on platelets, has been proposed as a promising alternative to stenting (149, 150). However, Abciximab is not approved for the management of cerebrovascular disease due to the negative results of previous studies (151) limiting its possible use in aPL-ischemic stroke.

#### Agents targeting inflammation

Several agents with anti-inflammatory and anti-oxidative properties may also prove beneficial in APS. Among them, one of the most studied is hydroxichloroquine (HCQ), which has been used for the treatment of SLE for many years. Available data show a potential role in the inhibition of toll-like receptors (TLRs) (152), and reduction of proinflammatory IL-1, TNF-α, and IL-2 (153). HCQ may also target the coagulation pathway by inhibiting platelet aggregation (154) and preventing the disruption of the annexin A5 shield by anti-β2-GPI antibodies (155). Interestingly, a 2013 non-randomized study by Schmidt-Tanguy et al. demonstrated a lower rate of recurrence of thrombosis in APS patients treated with oral anticoagulation plus HCQ vs. oral anticoagulation alone (156). Other potential agents include N-Acetylcysteine (NAC) and mitochondrial cofactor coenzyme Q10 (9). Statins, a class of HMG-CoA reductase inhibitors commonly used to treat dyslipidemia and atherosclerosis, have shown a potential to inhibit aPL-mediated thrombogenesis and modulate the proinflammatory milieu in APS, and both *in vitro* and *in vivo*, through the down-regulation of the expression of intracellular adhesion molecule 1 (ICAM1), vascular endothelial growth factor (VEGF) and of pro-inflammatory cytokines, including IL-1β, TNF-α, and interferon (IFN)-α (157, 158).

#### Monoclonal antibodies

Belimumab (BEL) is a monoclonal antibody directed against the B-cell activating factor (BAFF or BLyS) which promotes B-cell survival and differentiation, approved for unresponsive SLE. A report by Yazici et al. described its use in two primary APS patients with pulmonary and skin manifestations, respectively. One of the patients showed partial benefit while the other complete remission of symptoms, while aPL profile remained unchanged (159). On the contrary, a report by Sciascia et al. demonstrated disappearance of aPL in three patients with APS secondary to SLE treated with belimumab (160). Eculizumab is a monoclonal antibody directed against the C5 fraction of complement approved for the treatment of paroxysmal nocturnal hemoglobinuria and atypical hemolytic uremic syndrome. To date a series of case reports have been published regarding its use in APS, specifically in the setting of CAPS and post-transplantation renal thrombotic microangiopathy, with encouraging results (161–170).

#### Other agents

Sirolimus is a macrolide with inhibiting properties on the mammalian target of rapamycin (mTOR), a kinase involved in many signaling pathways related to cellular growth, proliferation, and survival (112). Previous studies have highlighted the possibility of the involvement of mTOR in the genesis of vascular stenosis in the context of endothelial injury (171, 172). Vascular cellular infiltrates and changes in the vessels intima and media layers have been observed in APS patients, especially in the context of CAPS (110, 111), suggesting a vasculopathic pathogenic mechanism. In a 2014 publication Canaud et al. described a series of patients with APS nephropathy undergoing transplantation. Interestingly, those receiving sirolimus had no recurrence of vascular lesions and showed decreased vascular proliferation on biopsy (173). Ongoing researches aim at identifying other therapeutic targets, including TLR 4 inhibition, TF inhibition, protease-activated receptor (PAR) antagonists, intracellular signaling blockers, and tolerogenic dendritic cells (142).

## Conclusions

APS is a rare and heterogeneous condition the neurologist may have to deal with. The diagnosis is not easy and a high level of suspicion must be held. APS should always be considered in young patients with CVA, especially in the absence of other vascular risk factors. Moreover, publications from the past decades have shown that many neurologic disorders not comprised in the original description by Hughes may harbor the disease. Elements such as autoimmune and connective tissue comorbidities, a positive history for obstetrical complications and refractoriness to standard therapy may guide the neurologist toward the correct diagnosis. A great amount of research aims at clarifying the complex etiopathogenesis of APS but many aspects remain obscure. The mechanism behind aPL-mediated pathology appears to be strongly, but not exclusively, thrombotic. Immune-mediated damage may be the key to some aPL-related neurologic manifestations. What favors the attack against nervous structures and what lies beneath the great phenotypic variability even among neurological presentations are open questions for researchers in the near future. On the one hand, mechanisms of immunological surveillance might be involved, as it was demonstrated in other more common forms of nervous system autoimmunity (174). On the other, genetic variability in neurobiological pathways may be worth careful investigation (76, 175). The double nature, thrombotic and immune-mediated, of APS is exemplified by current therapeutic strategies. Anticoagulation with warfarin represents at the moment the most effective therapy in the setting of thrombotic events. Anticoagulation has also been reported to reverse non-criteria neurologic manifestations that are not primarily thrombotic in their origin, further supporting the role of thrombosis in the pathogenesis of APS. On the other hand targeting the immune response through steroids, rituximab, IVIG or PE, can improve many non-criteria symptoms. A great number of other treatments are under investigation. DOACs represent an appealing alternative to warfarin, especially considering the young age of APS patients and the necessity of INR monitoring. Nevertheless, available evidence does not support their use. Other interesting options include antioxidant agents, monoclonal antibodies, and several agents targeting specific cells or molecules in the complex pathogenetic pathway of APS. One last interesting issues concerns the relationship between APS and SLE. A 2010 report by Veres et al. on a group of 165 patients with primary APS showed that 23% of cases converted to definite SLE within 10 years (176). The correlation between these two entities is not fully understood. The term neurolupus indicates the involvement of the nervous system in the setting of SLE. Pathogenic mechanisms, similarly to APS, include thrombosis, vascular proliferative changes and, possibly, anti-neuronal and anti-glial antibody activity (177). Further studies are needed to understand the relation between these two entities, shared mechanisms of nervous system injury and possible therapeutic options.

## Author contributions

All authors listed have made a substantial, direct and intellectual contribution to the work, and approved it for publication.

### Conflict of interest statement

The authors declare that the research was conducted in the absence of any commercial or financial relationships that could be construed as a potential conflict of interest.
